# Group-based psychoeducational workshop for parents in Kenya: findings from a pilot study

**DOI:** 10.3389/fpubh.2023.1223804

**Published:** 2023-09-15

**Authors:** Rediet Emebet Getnet Alemu, Hilda Nyatete, Rosine Baseke, Veronicah Ngatia, Tom L. Osborn, Christine M. Wasanga

**Affiliations:** ^1^Shamiri Institute, Addis Abeba, Ethiopia; ^2^Shamiri Institute, Nairobi, Kenya; ^3^Department of Psychology, Kenyatta University, Nairobi, Kenya

**Keywords:** psychoeducation, mental health literacy, family relationships, parents, adolescents, community-based

## Abstract

**Introduction:**

Low levels of mental health literacy amongst parents can have negative effects on youth mental wellbeing and help-seeking behaviors. Here, we explored the impact of a brief psychoeducational workshop on improving parent mental health literacy and family relationships in Kibera, a low-resource high-risk setting in Nairobi, Kenya.

**Methods:**

The workshop was designed to address this issue, and it was delivered by trained facilitators to small groups of parents (*N* = 72). Data was collected at baseline, post-workshop, two-week follow-up, and one-month follow-up.

**Results:**

Statistical and thematic analysis of the data revealed significant improvements in parent mental health literacy scores and family relationships, indicating the acceptability and effectiveness of this workshop.

**Discussion:**

The findings suggest that brief, group-based psychoeducational workshops can be effective in improving parent mental health literacy and family relationships, thereby addressing challenges faced by parents and youth in the Kenyan context. Future studies are needed to conclusively determine if such workshops can improve participants’ own mental health or their perception of child behavior.

## 1. Introduction

While 1 in 2 Kenyan adolescents report elevated symptoms of depression and anxiety, help-seeking remains limited due to factors like lack of accessible resources and societal stigma of mental illnesses (1).

Globally, low help-seeking behavior among youth has been associated with limited mental health literacy amongst parents (2). Mental health literacy can be defined as the “knowledge, attitudes, and beliefs about mental disorders and help seeking that can facilitate symptom recognition, management, and prevention” (2). Mental health literacy also includes a perceived ability to provide psychosocial support to others (2).

Parents function as crucial mental health caregivers (3). When faced with mental health problems, adolescents are more likely to approach their family and friends, especially their parents, than they are to approach mental health care services (4). In these instances, parents with increased mental health literacy are more likely to be able and willing to contact such services for their children (2). Unfortunately, mental health literacy remains low amongst parents worldwide (2), especially when children are in the vulnerable time of ages 13–18 (5).

Societal stigma affects the extent to which parents play a caregiving role for youths struggling with mental health problems (3). For instance, stigma is sometimes directed at and experienced by parents of adolescents struggling with mental health problems thus exposing parents to emotions of self-blame and guilt for their child’s problems (3). These emotions can impede the parents’ own wellbeing, parenting abilities, and how they view their child and their behavior (3). Such negative consequences are likely to be exacerbated for parents with low mental health literacy due to a low understanding of mental health, mental health disorders and coping strategies. This reality makes conditions even worse for adolescents —who are likely facing numerous stressors already (3).

Accordingly, researchers are starting to recognize that increasing parental mental health literacy could be integral to enhancing youth wellbeing (5) and help-seeking trends amongst youth (6, 7). Educational interventions —a set of activities intended to improve specified thoughts, emotions or behaviors of participants (8) — directed at parents have emerged as a result (7). These interventions are often brief, have multiple means of access and target parents of adolescents (2, 7). Moreover, these interventions commonly take a didactic approach with a focus on adolescent mental wellbeing and common mental health disorders amongst adolescents (2, 6, 7). Such interventions have proven effective in improving the youth’s help-seeking trends, and youth’s attendance at mental health interventions (9), in addition to enhancing youth wellbeing and parent–child relationships (5).

Given the rather vital role that parents play in the help-seeking process, it is important to develop interventions that increase parental mental health literacy. One such approach may involve psychoeducation. Psychoeducation is a practice which consists of psychotherapy and education (10). The psychotherapeutic components of this practice are reflected when participants engage in conversation with the intervention provider —a trained mental health practitioner— and with other participants, as though in group therapy (10). The educational components of this practice are reflected in the content of the discussions; the content can range from etiology of a specific mental illness to coping strategies and ways to improve one’s overall mental wellbeing (10). This method has been utilized in parent-targeting interventions and workshops — forums or gatherings which focus on thorough discussions between participants (11) — and has successfully improved participants’ mental literacy (2).

### 1.1. Objectives

For this pilot study, our team sought to develop and pilot a parent psychoeducational workshop in Kibera, a low-resource high-risk setting in Nairobi, Kenya. The primary objective of this study was to assess the impact of this workshop on participants’ mental health literacy. Additionally, we aimed to assess participants’ family conditions, mental wellbeing, and perception of their child(ren)’s mental health (as secondary outcomes). Our team hypothesized that all the primary and secondary outcomes would significantly improve as a result of attending this workshop; the null hypothesis stated that participating in this workshop will have no significant effect on the primary and secondary outcomes.

## 2. Methods

### 2.1. Study setting and design

The study was conducted at Shining Hope for Communities (SHOFCO) Girls’ School, a local nonprofit run school that serves students in the Kibera area. SHOFCO Girls’ School has a previous working relationship with our team at Shamiri Institute, a Kenya-based nonprofit dedicated to developing and disseminating youth mental health interventions. Since this school also works closely with parents in the community, the Parent Workshop was to be piloted in the SHOFCO campus located in Kibera, Nairobi. The SHOFCO team requested for all workshop materials and measures to be in simple Kiswahili as opposed to English, since parents are reportedly more comfortable with the former.

### 2.2. Participant recruitment

#### 2.2.1. Eligibility criteria

Parents of students in the participating school who have at least one adolescent child and reside in the Kibera area were eligible to participate in this study. No other inclusion or exclusion criteria were implemented.

#### 2.2.2. Recruitment

The SHOFCO team reached out to ~100 parents with an invitation to participate in this pilot. Parents were informed they would participate in a 2-h psychoeducational workshop which might improve their knowledge about adolescent mental health, in addition to improving their own mental health and family relationships. Parents were also informed they would each get KES 500 as travel reimbursement and participate in follow-up data collection.

To accommodate for parents’ busy schedules, the workshop was to be piloted in two cohorts: a morning cohort and an afternoon cohort. Parents were given the option to sign up for the one more convenient for them — with the total number of participants in each cohort kept below 50 for an even distribution amongst groups.

### 2.3. Procedures

**Figure 1:**
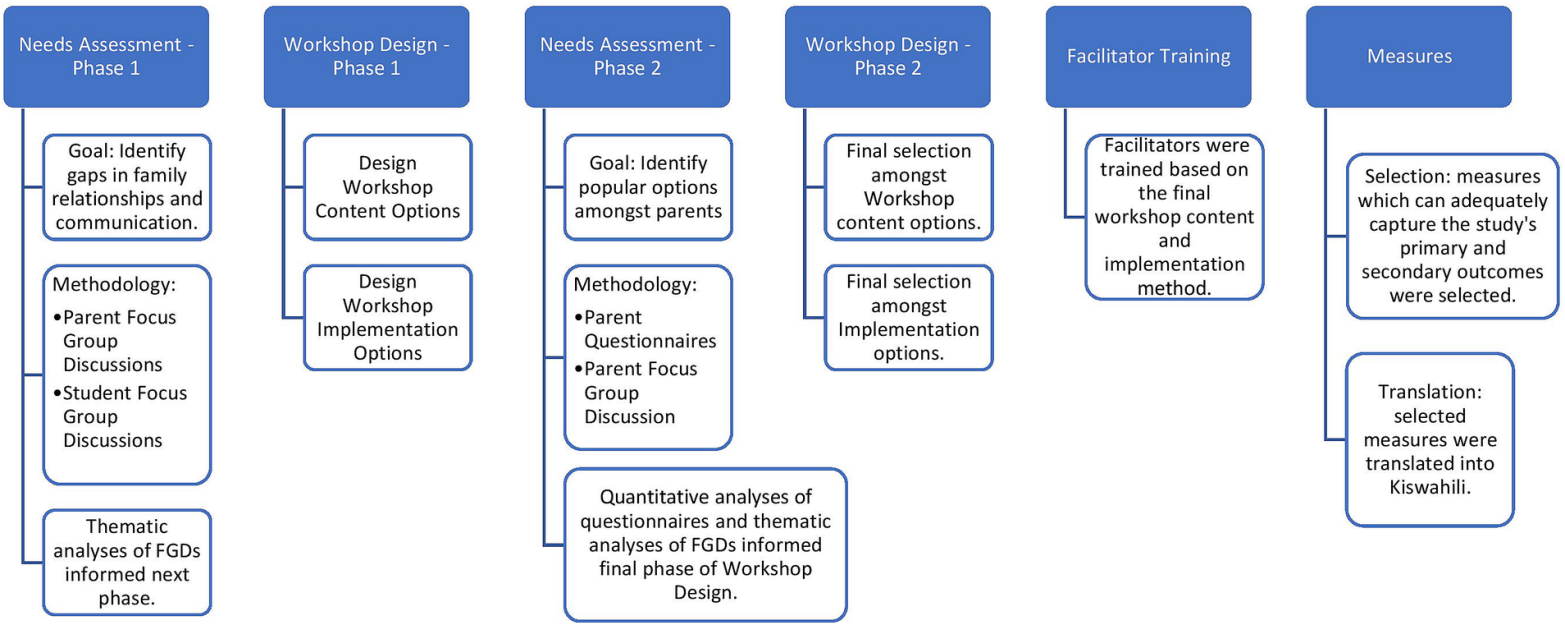
Diagram depicting the various stages of needs assessment and workshop design, in addition to facilitator training and selection and translation of study measures.

In developing the workshop, our team adopted an approach that included two phases of needs assessment and two phases of workshop design. After these phases were complete, the facilitators were trained based on the final workshop content and implementation methods. This was followed by selecting and translating the study measures, finalizing the preparations for implementation. See Figure 1 and following sections for further details.

Through the study, we were guided by the need of doing inclusive community – based research in the school context. As such, we included teachers, community – members, and our team was a majority local researchers all based in Kenya. These efforts enriched our research process strengthening our ability to develop a socio-culturally acceptable workshop.

#### 2.3.1. Needs assessment – phase 1

In recent years, our research team at Shamiri Institute has conducted the Shamiri Intervention amongst youth in Nairobi Kenya via different randomized-controlled trials (12, 13). This intervention is rooted in concepts of positive psychology — a branch of psychology which focuses on elements which support one’s wellbeing and ability to thrive (14) — and character-strengthening; it is a lay-provider delivered, group-based intervention which has been shown in previous trials to reduce depression and anxiety symptoms and improve a range of other psychosocial outcomes amongst Kenyan adolescents (12, 13).

Based on experiences of aforementioned research trials (12, 13), our team had observed significant gaps in communication between students and parents, students and teachers, and parents and teachers. Since social support — both perceived and objective — is critical to youth mental health (15), it was a priority to investigate these gaps and identify solutions to improve these lines of communication.

Accordingly, our team conducted a series of focus group discussions (FGDs) with secondary school students, parents, secondary school teachers, and school administrators. The students and school staff were selected randomly from schools which have previously worked with Shamiri Institute, while the parents were recruited by a Shamiri staff member via convenience sampling. Our team conducted and analyzed the qualitative data from all FGDs; the primary findings have been described below. See Supplementary material for more information.

##### 2.3.1.1. Student focus group discussions

The purpose of this set of FGDs was to get students’ perspectives on the following: (a) factors affecting youth wellbeing, (b) factors affecting the youths’ relationships with parents and schools and (c) new and existing solutions to improve youth wellbeing.

Across the student FGDs, the participants explained that the primary factor affecting their wellbeing is academic pressure, both from parents and teachers. Participants also explained that they feel as though parents do not trust them and neglect to acknowledge their own unique set of capabilities. Moreover, students expressed their desire for increased time spent with parents.

##### 2.3.1.2. Parent focus group discussions

The purpose of this FGD was to get parents’ perspectives on the following: (a) factors affecting youth wellbeing, (b) factors affecting parents’ relationships with their children and their schools and (c) new and existing solutions to improve youth wellbeing.

In the Parent FGD, barriers to family relationships — specifically, parent–child communication — were discussed extensively. Much of this discussion focused on parents’ wrongdoings, with an emphasis on not allowing individuality and vulnerability at home, “tyrannical parenting,” not having good parenting skills, and having unrealistic expectations for children. Participants also offered potential solutions to improve youth wellbeing highlighting the room for improvement amongst parents themselves. The most popular suggestions amongst participants included (1) parents allowing more individuality and listening to their children’s needs (instead of assuming what they are) and (2) increased access to resources for improving parenting skills.

#### 2.3.2. Workshop design – phase 1

Based on the findings of the first phase of needs assessment, our team decided to develop parent-targeted holistic psychoeducation content to be delivered in the form of a group-based workshop. Several options — described briefly below — would form the basis of a second phase of needs assessment to highlight the most practical and desired options amongst parents.

##### 2.3.2.1. Workshop content options

*Module 1: introduction*. This module focused on the importance of parental emotional support for youth mental health. It also included data reporting on the correlation of Kenyan adolescent mental wellbeing and perceived social support from family (15), complemented by a definition of mental wellbeing. The purpose of this discussion was to highlight the need for providing emotional support for the youth. While this concept might be intuitive for parents, some researchers have stressed that this reminder is nonetheless quite helpful for parents to fully realize that the onus of sustaining good mental health is not solely on the youths themselves (5).

*Module 2: Kenyan youth mental health*. This module provided explanations of the current state of youth mental health in Kenya. Prevalent mental health problems were to be discussed along with how these problems can affect youths’ daily lives and wellbeing. This module also explained how to differentiate between common adolescent behaviors and symptoms of mental health problems. The purpose of this discussion was to inform parents about different elements of youth mental health to encourage them to interact more with their children and listen more actively to them.

*Module 3: Shamiri intervention*. This module covered the basic psychological concepts (growth mindset, gratitude, and value affirmation) and implementation methods which make up the Shamiri Intervention (13). These explanations were to be followed by a discussion on how to incorporate the practice of these concepts into family relationships. The goals of this module were to encourage participants to interact more with their children and to introduce them to parenting techniques rooted in positive psychology and encouragement — techniques proven to be more beneficial to youth wellbeing when compared to control and punishment (16). This module contributes to the uniqueness of this study as other (psycho)educational workshops for parents often focus on general adolescent mental health and common mental health problems (2, 6, 7).

*Module 4: psychological first aid*. This module explained the purpose and procedures of Psychological First Aid and scenarios in which it might be necessary to carry out this process. There was an emphasis on the fact that this procedure can be used by anyone — without formal counseling training — for a person experiencing a distressing event. This lesson also included an explanation of confidentiality and its importance for instances during which any parent is providing PFA for individuals who are not their children. The goal of this module was to improve participants’ skills and self-confidence in how they respond to distressing events, especially those experienced by their children. Additionally, this module was intended to emphasize the necessity of confidentiality and instances in which individuals are allowed to break confidentiality.

##### 2.3.2.2. Workshop implementation options

*Option A: virtual workshop*. In this option, the workshop would be delivered digitally in the form of self-guided reading materials and/or audio recordings. After being granted access to these resources, participants would have 2 weeks to complete the workshop.

*Option B: hybrid workshop*. In this option, the workshop would still be delivered digitally, as explained in Option A. However, upon their completion of the virtual workshop, participants would be invited to join groups of 10–15 other parents in discussion and activities on the workshop content. Each group would be moderated by a trained facilitator.

*Option C: hybrid workshop*. In this option, participants would first be invited for in-person group discussions and activities on the workshop content. After the group discussions, participants would be granted access to a virtual workshop. This is to encourage them to review the concepts they will have discussed already.

*Option D: in-person workshop*. In this option, participants would be invited for in-person group discussions and activities. There will be no virtual components before or after these discussions.

#### 2.3.3. Needs assessment – phase 2

Since the first phase of workshop design yielded multiple options for workshop content and implementation method, this phase of needs assessment was intended to identify the options which are most popular amongst Kenyan parents of adolescents. In terms of workshop content, the introductory module was not included in this phase of needs assessment as the study team agreed on its crucial role to set up the discussion for the remaining modules.

Initially, our team sought to collect data from parents who represent the general Kenyan population. Accordingly, we arranged to work with Alliance High School (AHS), a national boarding high school with students from different parts of the country. Initially, our team designed brief questionnaires which consist of the options described in Section 3.2. and arranged with AHS to distribute them to parents during an Annual General Meeting. Based on the data collected from ~150 parents, it was evident that the most popular options were the Shamiri Intervention module and the first of the hybrid workshops.

To supplement the feedback from the general population, our team set up an FGD to collect data from parents in a similar demographic to our target population. The FGD participants were randomly recruited amongst parents whose children attend Elite Visionary High School —a non-boarding high school located in Kibera.

In this FGD, members of the study team explained the purpose of the workshop and presented the different options of implementation and content to the participants for discussion. The findings from this FGD strongly indicated that the Shamiri Intervention Module was the most popular as opposed to the psychoeducation and psychological first aid modules, similar to the findings from the general sample. In terms of implementation method, participants preferred the in-person implementation method. Here, the findings varied from those of the general sample. However, the study team decided to proceed with the in-person implementation method considering that it was the preference of the target population sample and the limited access to smart phones and inadequate funds for internet access amongst this population. See Supplementary material for more information on this phase of needs assessment.

#### 2.3.4. Workshop design – phase 2

According to the findings from the second phase of needs assessment, the workshop content was modified to prioritize the Shamiri Intervention Module and to be delivered in-person via trained facilitators. Moreover, the workshop content was designed to be discussion-heavy to boost participation from parents. The workshop protocol is available amongst the Supplementary material for further reading.

#### 2.3.5. Facilitator training procedures

##### 2.3.5.1. Facilitator recruitment

The facilitator role for this project entailed communicating the purpose and components of the workshop to the participants, supporting participants to complete the questionnaires, effectively guiding group discussions and encouraging fair participation. Moreover, while the workshop session protocol was prepared in English, facilitators were responsible for presenting the discussions in a form of Kiswahili with which their group was most comfortable.

Amongst the Shamiri Institute staff, five associates who had a bachelor’s degree in a mental health-related field were selected as facilitators. These associates had previously been trained and worked as clinical supervisors for the dissemination of the Shamiri Intervention, which is described in a previous section. As Shamiri supervisors, they had already had experience in training and supervising lay providers of the intervention, handling clinical cases arising from intervention participants, and developing session protocols for different programs. Moreover, all associates were fluent in both Kiswahili and English.

##### 2.3.5.2. Facilitator training

An eight-hour training was delivered by the PI and one of the co-authors. The initial reading materials included the project proposal, the workshop protocol, the baseline and endpoint questionnaires (both English and Kiswahili versions), and an article describing the network approach to psychopathology (17).

The training was divided into two sessions: didactics and roleplays.

*Didactics*. The purpose of this session was to discuss the reading materials and highlight the intended outcomes of the project. First, the PI led a discussion on the project proposal. Then, the PI delved into the network approach to psychopathology, to supplement the reading with discussions on its potential benefits to the project. Here, it was critical to encourage facilitators to avert their focus from clinical diagnostic labels towards the specific problems experienced by individuals. This was done to make the workshop content and discussions more direct and culturally acceptable for participants.

In the second half of this session, the PI went through each element of the workshop session protocol and explained the reasoning behind each element’s curation and how it contributes to the workshop’s overall objectives. This was followed by a discussion on the objectives of the different measures and a run-through of the schedule for the day of the pilot.

*Roleplays*. After the first session, the remaining 5+ h were dedicated for roleplays and discussions. Each facilitator was given 30 min to practice while using the workshop protocol by facilitating a group of the other facilitators and one of the co-authors, who were all acting as participants. Prior to the start of the roleplays, the facilitators were reminded to utilize basic skills of peer counseling —such as validation, summarizing, open ended questions, etc. — and group leadership techniques —such as keeping participants from going off topic, time management, engaging quiet participants, etc. After each facilitator’s roleplay session, they were asked to identify areas in which they performed well and those which could use improvement. The remaining facilitators then offered their own views of the facilitator’s performance. Finally, the co-author provided constructive feedback.

At the end of this session, the facilitators were reminded to keep reading through the workshop session protocol prior to the implementation date. Any potential difficulties experienced were to be communicated to either the PI or the co-author for clarification and assistance. Facilitators were all instructed not to stray from the workshop session protocol to ensure similar flow of discussion between the different groups and reduce bias.

### 2.4. Outcomes

The primary outcome for this study was parent mental health literacy. The secondary outcomes were participants’ family conditions, mental wellbeing, and perception of their child(ren)‘s mental health. The study team selected the following questionnaires in order to collect participant data that can allow us to measure these outcomes.

### 2.5. Measures

#### 2.5.1. Primary outcome measure

##### 2.5.1.1. Parent mental health literacy questionnaire

The PMHLQ is a 12-item questionnaire designed by the PI to assess parent mental health literacy amongst participants in this workshop. Factors integral to mental health literacy -such as one’s knowledge about mental health and mental health problems, attitude towards people who have mental health problems and perceived ability to help others (2), are included in this questionnaire, with most questions examining these factors from a parent’s perspective. This questionnaire was influenced by the Mental Health Literacy Scale (MHLS) which was designed to measure one’s level of information and perceptions regarding mental wellbeing and mental health problems (18).

#### 2.5.2. Secondary outcome measures

##### 2.5.2.1. Satisfaction with family life

The SWFL was built upon the Satisfaction with Life scale in order to provide a more flexible and inclusive set of questions to measure how one views one’s family life (19). While we have not found prior studies which have validated the SWFL in Kenyan settings, there is ample evidence that this scale can maintain its validity amongst different cultural settings and varying family structures (19).

##### 2.5.2.2. Patient health questionnaire 8-item

The PHQ-8 is a modified version of the PHQ-9, a questionnaire which assesses individuals’ depressive symptoms (20). The PHQ-8 was used in order to omit the ninth item of the PHQ-9 which focuses on suicidal thoughts and behaviors. This is because suicide is a heavily stigmatized topic amongst different Kenyan and African populations (13). Otherwise, research studies have validated and successfully used this questionnaire in our context (13).

##### 2.5.2.3. Generalized anxiety disorder 7-item

The GAD-7 measures individuals’ anxiety symptoms (21). This questionnaire has been validated amongst different Kenyan populations (13).

For the project, the PHQ-8 and GAD-7 were merged — with each question appearing as an option — and the participants were asked to mark the checkbox for each option that has negatively affected their work, family, and social lives. In a follow-up question, participants were asked to select 2–3 of the options that have the most significant negative impact on work, family, and social lives. These modifications were made to identify the specific problems affecting participants’ mental health and to reduce the time spent on completing the questionnaires.

##### 2.5.2.4. Short Warwick-Edinburgh mental wellbeing scale

This 7-item questionnaire aims to assess individuals’ mental wellbeing (22). It has been validated amongst different groups across the world (22).

##### 2.5.2.5. Child behavior checklist

The CBCL is designed to assess the presence of mental health problems amongst children aged 6–18 via parents’ or guardians’ observations (23). We will provide a modified version of it to parents before the start of the parent workshop and at follow-up data collection. The CBCL has been effectively used in various Kenyan and other LMIC settings –in its original English version and a translated Kiswahili version (24).

#### 2.5.3. Acceptability and feasibility

##### 2.5.3.1. Workshop evaluation questionnaire

The study team developed this questionnaire to evaluate the efficacy and practicality of the workshop. It included questions regarding the delivery, content, and timing of the workshop and the expectations for participants.

#### 2.5.4. Translation

First, all study measures were translated from English to Kiswahili by two forward translators, both Shamiri Institute staff. Then, the translation committee — comprised of three Shamiri staff, all fluent in both languages, two of which are mental health professionals — reviewed the Kiswahili questionnaires and met with the forward translators to discuss the necessary changes. The forward translators then modified the Kiswahili questionnaires and met with the committee to review the revised draft. Afterwards, two back-translators — both Shamiri Institute staff who had not interacted with the original English questionnaires — received the Kiswahili questionnaires and translated them back into English. The translation committee and forward translators then met to compare the original English questionnaires with the back-translated versions to identify areas of modification in the Kiswahili questionnaires. After the necessary modifications were made, the final versions of the Kiswahili questionnaires were ready for use.

#### 2.5.5. Data collection plan

The team collected data at four timepoints: beginning of the workshop, end of the workshop, two-week follow-up and one-month follow-up.

The participants completed baseline questionnaires at the beginning of their workshop session. This questionnaire consisted of all the measures earlier. At the end of the workshop sessions, participants completed endpoint questionnaires which consisted of the PMHLQ and Workshop Evaluation Questionnaire.

For the two-week follow-up data collection, three participants were randomly selected from each facilitator’s workshop groups. Prior to the two-week mark, the group facilitators called the participants to schedule interviews; all participants had already been informed of the random possibility of receiving a phone interview at that time. All interviews were conducted using a guide developed by the study team to ensure similar interview structures and to reduce bias.

For the one-month follow-up data collection, all participants were asked to come to the SHOFCO campus. The facilitators met with their initial groups and supported the participants to complete the questionnaires which included all the measures earlier.

#### 2.5.6. Data analysis plan

##### 2.5.6.1. Qualitative data analysis plan

First, an interview guide was prepared by the PI and translated into Kiswahili by the workshop facilitators. The purpose of the interview guide was (1) to determine if and how participating in the Shamiri Parent Workshop affected the participants and their family lives, and (2) to ensure that the questions were uniform across all the interviews.

While one facilitator was conducting an interview, another facilitator was present to note down the participants’ responses. These notes were translated into English and categorized per participant and per question before being compiled into one file by the facilitators. Then, the PI developed a code hierarchy which categorized all the codes — a representative of each distinct response collected from the phone interviews — primarily into themes which are representative of a question in the interview guide. Similar codes within each theme were further categorized into sub-themes to facilitate more effective analysis, with few of the sub-themes doubling as codes. Then, the code hierarchy was transferred to an excel sheet, on which the PI and a member of the study team recorded the frequency of each code independently. The two excels were then compared, consolidated into one file, and used to analyze the interview data by highlighting the codes and sub-themes with the highest frequencies within each theme.

##### 2.5.6.2. Quantitative data analysis plan

All analyses were done on R Studio (version 4.3.0). Missing data were analyzed using the ‘mice’ package to create multiple imputations through predictive mean matching. We used an imputed dataset for all subsequent analyses.

###### 2.5.6.2.1. Descriptive analyses of baseline data

Descriptive analyses were conducted using the psych package in R, calculating the means and standard deviations of all measures. To make comparisons between the mean scores for the groups, the data was grouped by sex, marital status, age, and educational level. Differences between the mean scores of all measures were arrived at using a simple statistical test (*t*-test) (25) in R.

###### 2.5.6.2.2. Differences between baseline and endpoint data

Different tests were conducted to test for differences between the means at different timepoints. For baseline and endpoint, a one-way ANOVA test was conducted.

###### 2.5.6.2.3. Differences between baseline and one-month follow-up data

To compare for differences at one – month follow-up, we ran a linear mixed effect model, implement using the ‘lmer’ function from the ‘lme4’ package. The outcome variable was modeled as a function of Time, with random effects for Group Leader (i.e., group facilitator) and for Participant (done to capture different trajectories for different individuals) using data from baseline to one-month follow-up.

###### 2.5.6.2.4. Differences between all timepoints

Subsequently, a multiple comparisons analysis was conducted, to determine which timepoints significantly differ from each other. The Tukey’s Honest Significant Differences (HSD) (26) was used for the multiple comparisons analysis.

#### 2.5.7. Workshop implementation

At the beginning of implementation, the study team introduced the goal of the workshop to the participants in the morning cohort. The team had prepared an on-site randomization tool with small ballots containing names of one of the facilitators. Each participant picked a ballot at random and was assigned to the facilitator whose name they chose. This was done to ensure that potential confounding variables such as age, gender and educational status were randomly distributed amongst the different groups.

After randomization, all groups started with personal introductions and proceeded to the baseline questionnaires. Participants who were stand-ins for the invited parents and who did not have adolescent children of their own were asked not to complete the questionnaire but remained throughout the workshop. All morning cohort workshop sessions were completed within 3 h.

After the morning cohort workshop sessions, the study team and facilitators met for a debrief. The facilitators explained that the groups took between 45 min and 70 min to complete the questionnaires, as some participants could not read and/or write and others mentioned that the Kiswahili used in the questionnaires was too formal. Accordingly, the team decided that the facilitators would read the questions aloud for the afternoon cohorts to support participants who could not read and to reduce the time taken to complete questionnaires.

For the afternoon cohort participants, the study team followed the above-mentioned steps of program introduction and on-site randomization. All workshop sessions were completed within two and a half hours.

## 3. Results

### 3.1. Demographics

The study sample consisted of 72 Kenyan parents of adolescents, of which 53 (73.6%) were female and 15 (20.8%) were male. A few participants (*n* = 4) did not answer some questions and resulted in 5.6% missing data on gender. The mean age of participants was 41 years (sd = 7.99). Of these participants, 44.4% reported being married and 48.6% had attained primary school as the highest level of education. See Table 1 for sample characteristics.

**Table 1:** Sample characteristics.

Characteristic		Mean *N* (%)	SD	Missing *N* (%)
Age		41.15	7.997	0
Sex	Female	53 (73.6)		4(5.6)
	Male	15 (20.8)		
Marital status	Single	4 (5.6)		13(18.1)
	Married	32 (44.4)		
	Separated	12 (16.7)		
	Divorced	11 (15.3)		
Highest level of education	Primary school	35 (48.6)		9(12.5)
	Secondary school	21 (29.2)		
	Diploma	4 (5.6)		
	Bachelor’s degree	2 (2.8)		
	Multiple degrees	1 (1.4)		

### 3.2. Results at endpoint (immediately post-workshop)

#### 3.2.1. Primary outcome

##### 3.2.1.1. Mental health literacy

To assess parents’ knowledge of mental health and mental health problems, the Parent Mental Health Literacy Questionnaire (PMHLQ) was used. The scale consisted of statements about mental health, mental health problems and general thoughts on the role of parents in children’s mental health. Participants were asked to rank on a Likert scale of 1 (strongly disagree) to 7 (strongly agree). Scores can range from 12 to 84, with higher scores indicating better understanding of mental health and mental health problems. The PMHLQ had low internal consistency, with a Cronbach’s alpha of 0.62. This can be attributed to the small sample size and the fact that it was being used for the first time in this population.

At baseline, participants had a mean score of 49.55 (SD = 10.2). At the end of the Workshop, participants completed the same questionnaire, and scored a mean of 54.75 (SD = 9.6). Statistical tests revealed that this increase was significant (*p* = 0.03166).

Participants showed the highest improvement (mean difference = +1.3) from the beginning of the workshop to the end, when asked whether they understood what is meant by mental health and mental health problems. There were no improvements on whether parents think people are born with a set of characteristics that do not change. See Table 2 for average scores for each question and the differences between baseline and endpoint scores.

**Table 2:** Mean score of items on the parent mental health literacy scale.

MHLS items	Baseline	Endpoint	Difference
When adolescents feel emotionally supported by their parents, they are likely to have better mental health	5.2	5.5	0.3
Parents should support adolescents to manage their mental health problems	6.1	6.4	0.3
I understand what is meant by mental health and mental health problems	4.8	6.1	1.3
A mental illness is a sign of personal weakness*	4.5	4.4	−0.1
I know the difference between common adolescent behaviors and signs of mental health problems	4.5	5.5	1
If I had a mental illness, I would not tell anyone*	2.6	1.6	−1
I have to be a professional to help someone experiencing a mental health emergency*	3.1	2.4	−0.7
If someone confides in me about their mental health problems, I would share this information with friends and family*	2.6	2.8	0.2
If my child told me she/he is experiencing mental health problems, I would know how to help	5.4	6.2	0.8
People are born with a set of characteristics that do not change*	2.4	2.4	0
Practicing gratitude can improve one’s mental health	4.8	5.3	0.5
Punishing bad behaviors is more effective than encouraging and rewarding good behavior*	4.4	4.0	−0.4

*Represents questions that were reverse coded. A lower mean score indicates positive mental health literacy. A negative difference in means on these questions indicates improvements from baseline to endpoint.

Male participants experienced the least change (+0.2) compared to their female counterparts (+7.87) on the mental health literacy scale between baseline and the end of the workshop. Older participants had lower mental health literacy scores, both at baseline and at the end of the workshop. This difference was significant (*p* = 0.0377).

#### 3.2.2. Acceptability and feasibility

##### 3.2.2.1. Program evaluation

Participants completed a set of questions about their thoughts on the workshop, selecting the option that reflected their experiences. They also rated some statements about the workshop on a scale of 1 (Strongly disagree) to 7 (Strongly agree).

Recommending the workshop to a friend was rated highly by participants when compared to the other items on the program evaluation questionnaire. When asked to select what they liked most about the workshop, learning about life skills was the most selected among participants (n = 40), followed by socializing/positive interactions (n = 15). Participants (n = 35) expressed their interest in increasing the length and frequency of sessions as ways of improving the Workshop. See Table 3 for ratings of program evaluation questionnaire at endpoint.

**Table 3:** Program feedback ratings at endpoint.

	Mean score (*n*)	Standard deviation
This workshop was helpful	6.2 (69)	1.6
I would recommend this workshop to a friend	6.45 (66)	1.2
This workshop was confusing	2.1 (63)	1.8
I enjoyed participating in this workshop	6.4 (61)	1.4
I am clear about what is expected of me as a result of going through this workshop	6.2 (67)	1.2
The facilitators are sufficiently skilled to lead this workshop	6.5 (67)	1.1
I will apply the lessons from this workshop in my family life	6.4 (68)	1.3
The time allocated to this discussion was enough	5.9 (67)	1.5

### 3.3. Results at two – week follow-up

As discussed earlier, the qualitative data from the two-week follow interviews was thematically analyzed by the study team. All interviewees’ distinct responses were identified as independent codes and categorized within a theme — equivalent to a question in the interview guide. Similar responses (codes) were then further classified into sub-themes.

In this section, all identified sub-themes — within each theme — are listed in descending order of popularity amongst interviewees. This is followed by an explanation of the most popular codes within the most popular sub-themes.

#### 3.3.1. Theme 1: interviewees’ favorite element of workshop

Interviewees’ answers were categorized into the following sub-themes: (1) the overall experience; (2) learning about children; (3) general learning and (4) gaining personal satisfaction. Within the first and most popular sub-theme, interviewees highlighted the elements of speaking with other parents and feeling heard. Within the second sub-theme, interviewees emphasized lessons on properly caring for children and properly communicating with children.

#### 3.3.2. Theme 2: interviewees’ least favorite element of workshop

Interviewees’ answers were categorized into the following sub-themes: (1) N/A and (2) different elements of the implementation method. Within the first sub-theme, most of the interviewees stated that they did not have any areas of dissatisfaction. Within the second sub-theme, interviewees explained that insufficient time was allocated to the Workshop, and they did not have enough time to hear from other parents.

#### 3.3.3. Theme 3: lessons learnt from the workshop

Interviewees’ answers were categorized into the following sub-themes: (1) how to improve intra-family relationships, (2) (importance of) understanding children, (3) lessons on mental health, (4) gentler methods of discipline and (5) miscellaneous lessons. Within the first sub-theme, interviewees explained that they have gained lessons on how to guide and support their children, in addition to having a better understanding of parenting. Within the second sub-theme, interviewees emphasized having learnt the importance of understanding their children’s experiences and challenges.

#### 3.3.4. Theme 4: ways of applying lessons learnt from the workshop

Interviewees’ answers were categorized into the following sub-themes: (1) increased positive involvement with children, (2) affirmative answers to having applied lessons learnt, (3) increased involvement with community, (4) negative answers to having applied lessons learnt and (5) increased discipline. Within the first sub-theme, interviewees explained that they have been actively listening to and have been more understanding of their children’s needs. Similarly, they stated that they have been interacting and communicating more with their children. Within the second sub-theme, almost all interviewees confirmed that they have actively applied the lessons they learnt from the workshop.

#### 3.3.5. Theme 5: impact of application of lessons learnt

Interviewees’ answers were categorized into the following sub-themes: (1) improved intra-family relationships, (2) affirmative answers to the presence of positive impact, and (3) personal satisfaction. Within the first sub-theme, interviewees testified to having better and more conversations with their children in addition to establishing a clearer understanding with their children. Interviewees also emphasized that they have noticed positive changes in their children’s behavior and experienced less intra-family conflict. Within the second sub-theme, nearly all interviewees confirmed that their lives — either personal, family or both — have been positively impacted by their application of lessons learnt from this Workshop.

#### 3.3.6. Theme 6: willingness to participate in a similar workshop

All interviewees stated that they were willing to participate in a workshop such as this one.

#### 3.3.7. Theme 7: additional lessons suggested by parents

Interviewees’ answers were categorized into the following sub-themes: (1) parenting, (2) coping strategies, (3) marriage-focused and (4) miscellaneous. Within the first sub-theme, interviewees emphasized that they would like additional lessons on how to communicate with and discipline children. Within the second sub-theme, interviewees mentioned they would like additional lessons with family issues, anger management and gender-based violence.

### 3.4. Results at one-month follow-up

#### 3.4.1. Parent mental health literacy

The model predicting self-reported parent mental health scores revealed significant effects for Time but nonsignificant effects for Age, Gender, Educational Level and Marital Status. The significant effect for time revealed significant improvements in parents’ mental health literacy scores from baseline – to – 4-week follow-up (*p* = 0.026; d = 0.31; Table 4).

**Table 4:** Parental mental health literacy model predicting self-reported parent mental health scores revealed significant effects for time.

Predictors	Mental health literacy			Estimates	CI	*p*
(Intercept)	50.01	39.48–60.54	**<0.001**
Time [baseline]	4.79	2.03–7.55	**0.001**
Time [1 month follow-up]	3.16	0.39–5.92	**0.026**
Age	−0.10	−0.32 – 0.12	0.384
Gender [male]	−2.94	−7.06 – 1.18	0.161
Educational level	0.01	−1.94 – 1.96	0.993
Marital status [married]	4.66	−2.19 – 11.52	0.181
Marital status [separated]	6.97	−0.47 – 14.42	0.066
Marital status [divorced]	3.30	−4.22 – 10.82	0.388

Random effects	
σ^2^	70.35
τ_00 Shamiri_ID_	24.30
τ_00 Group_Leader_	1.91
ICC	0.27
N _Group_Leader_	5
N _Shamiri_ID_	72
Observations	216
Marginal *R*^2^/Conditional *R*^2^	0.084/0.333

The bold values indicate statistical significance where the *p*-value is <0.05.

#### 3.4.2. Parent mental wellbeing

The model predicting parent wellbeing revealed significant effects for the covariates age and marital status but not Time, Gender, or Educational Level (Table 5).

**Table 5:** Results for parental wellbeing and satisfaction with family life models.

Predictors	Parental wellbeing			Satisfaction with family life	Estimates	CI	*p*	Estimates	CI	*p*
(Intercept)	23.02	16.40–29.63	**<0.001**	16.73	9.59–23.87	**<0.001**
Time [1 month follow-up]	0.55	−0.87 – 1.97	0.444	2.74	1.13–4.35	**0.001**
Age	−0.17	−0.30 – −0.03	**0.015**	−0.04	−0.19 – 0.10	0.571
Gender [male]	1.67	−0.93 – 4.27	0.205	0.09	−2.73 – 2.91	0.950
Educational level	−0.30	−1.53 – 0.93	0.632	−0.27	−1.59 – 1.06	0.690
Marital status [married]	3.57	−0.87 – 8.01	0.114	−1.70	−6.51 – 3.11	0.487
Marital status [separated]	4.86	0.13–9.59	**0.044**	−2.11	−7.23 – 3.02	0.417
Marital status [divorced]	3.54	−1.25 – 8.33	0.146	−2.72	−7.93 – 2.48	0.302

Random effects		
σ^2^	18.35	23.55
τ_00 Shamiri_ID_	9.66	10.05
τ_00 Group_Leader_	0.00	0.00
N _Group_Leader_	5	5
N _Shamiri_ID_	72	72
Observations	144	144
Marginal *R*^2^/Conditional *R*^2^	0.120/NA	0.095/NA

The bold values indicate statistical significance where the *p*-value is <0.05.

#### 3.4.3. Satisfaction with family life

At one-month follow-up, the model predicting parent satisfaction with life predicted significant effects for Time but not Age, Gender, Educational Level or Marital Status. This significant effect for time revealed significant improvements in parental self-report family life satisfaction scores from baseline – to – 4-week follow-up (*p* = 0.001; d = 0.35; Table 5).

## 4. Discussion

The purpose of this study was to design and pilot a psychoeducational workshop which can improve parents’ mental health literacy, in addition to their family relationships, mental wellbeing and perception of their children’s behavior. The study utilized a grassroots approach to ensure that the content and implementation method of this workshop were primarily informed by the findings of the needs assessment phases (conducted with local adolescents and parents of adolescents). This approach contributes to the uniqueness of the study as it is likely to ensure the study design is tailored for this specific context. The workshop was discussion-heavy and focused on concepts of character strengthening and positive psychology from the Shamiri Intervention, with conversations on the importance of parental emotional support to adolescents, Kenyan youth mental health and psychological first aid. This workshop was delivered by local trained psychologists with further training on the implementation method and content of this workshop.

### 4.1. Parent mental health literacy

Using the in-house scale PMHLQ, participants’ mental health literacy scores showed statistically significant improvements at endpoint (post-workshop). At the two-week follow-up, participants stressed that they have learnt of the importance of understanding children’s experiences. Finally, participants’ scores at the one-month follow-up show statistically significant improvements, when compared to baseline data. All three results indicate the participants’ mental health literacy improved after attending the workshop.

Improvements in parental mental health literacy can help participants better understand and support their children (2, 5, 7), alleviating significant challenges that arose in the first phase of our needs assessment. Parents had highlighted that most parents do not allow individuality and vulnerability in their homes and do not possess effective parenting and communication skills with their children. Students shared concerns that parents do not trust them and neglect to get to know their personalities. The improvements in parent mental health literacy reported by this study’s participants have evidently resulted in parental behavior changes that are likely to address these concerns. These improvements can also be integral to foster help-seeking for youth mental health problems by enhancing parents’ willingness and ability to recognize these problems and contact mental health care providers (6, 7).

### 4.2. Family relationships, parent mental wellbeing, and perception of child behavior

#### 4.2.1. Intra-family relationships

From the two-week follow-up data, it is evident that participants had learnt lessons on improving family relationships and implemented these lessons by being more involved with their families. As a result, participants also reported experiencing improved family conditions including improved child behavior and less intra-family conflict. Similarly, the one-month follow-up data reveals that participants’ scores on the SWFL scale increased significantly when compared to baseline, supporting findings from other parent-focused workshops (5).

The reported improvements in family conditions and communication address parental challenges and needs identified in the first phase of our needs assessment. Moreover, parents who feel more equipped to openly communicate with their children are likely more capable of fostering trust within the family, addressing the primary concern that arose during the student FGDs — lack of trust.

Our findings which depict that participants have increased their involvement in their (adolescent) children’s lives are quite important considering the significant impact of social support on youth mental health. Previous studies, conducted in this context, show that social support (from family and friends) has a statistically significant negative correlation with youth mental health problems (1, 15). Accordingly, improving parental support can be a means of improving youth mental health.

#### 4.2.2. Parent mental wellbeing

The two-week follow-up data shows that participants experienced a positive impact in their personal lives. However, this data did not reflect specific improvements in participants’ mental health. During the one-month follow-up, there were no significant improvements on the SWEMWBS. Moreover, the formatting of the questions of the PHQ-8 and GAD-7 proved to be confusing to participants and failed to yield reliable data. Therefore, there is no conclusive evidence that attending this workshop improved participants’ mental wellbeing (or reduced symptoms of mental health problems). In future trials, incorporating activities which allow parents to practice the concepts of the Shamiri Intervention for their personal wellbeing — instead of solely in their relationship(s) with their child(ren) could yield significant improvements in parent mental wellbeing.

#### 4.2.3. Perception of child behavior

The format of the CBCL was reported to be confusing to participants. Both participants and facilitators stated that the (translation of the) questionnaire did not provide sufficient instructions as to which of their children to keep in mind while completing this questionnaire. Some participants also expressed that they completed the questionnaire with all their children in mind. As a result, our team was unable to use the data from the CBCL to determine the workshop’s impact on the participants’ perception of their child(ren)‘s behavior. In future trials, the study team will work with a sample of the target population to identify and apply the necessary modifications to the CBCL.

With that said, our team does have qualitative data indicating that participants reported an increased understanding of the importance of listening to and understanding children and their challenges.

### 4.3. Acceptability and feasibility

Participants’ responses to the program evaluation questionnaire showed that the workshop was enjoyable, understandable, and helpful. Participants also appreciated the facilitators’ skillset. At one-month follow-up, participants expressed strong commitment to applying lessons learnt in their daily/family lives and enthusiasm for recommending the workshop to friends. These results indicate that the workshop was a valuable and sensible experience for the participants, making it an acceptable form of workshop for parents in this context.

Here, it is crucial to note the importance of conducting needs assessment to design and test, modify, and disseminate tools which are acceptable and feasible in the local context. This is particularly important in African contexts, contexts in which most such studies are led by western researchers and conducted without appropriate adoption to the local context (27). Centering the insights of the local population can also enhance a given study’s efficacy and integrity (27).

### 4.4. Conclusion

Parent mental health literacy significantly impacts youth mental health and help-seeking trends. Intra-family relationships and communication, in addition to parent mental health, are also critical to fostering youth mental health. Accordingly, our team designed a brief workshop — in the form of a group-based psychoeducational workshop — to improve parent mental health literacy, family relationships, parent mental wellbeing and perception of child behavior. This workshop was facilitated by local clinical psychologists and lasted around 3 h. As a result of attending this workshop, participants reported statistically and qualitatively significant improvements in their mental health literacy scores and family relationships. Participants also reported that the workshop was enjoyable and helpful, suggesting the acceptability of this workshop. Unfortunately, there was no conclusive evidence to suggest that this workshop improved participants’ own mental health or their perception of child behavior. Regardless, these findings strongly suggest that brief psychoeducational workshops can be effective in improving parent mental health literacy and family relationships, alleviating challenges highlighted both by parents and youth in the Kenyan context.

### 4.5. Limitations

Even though the participants’ PMHLQ scores improved significantly after the workshop, this scale was developed for the purpose of this study and has yet to be tested for its reliability and validity within other contexts.

The workshop was intended to last up to 2 h, at most, but some sessions ran for as long as 3 h. This increase in duration can reduce the scalability and feasibility of this workshop within other contexts. A significant contributor to the increased duration of the workshop was the challenges faced by participants when completing the questionnaires, such as difficulties in reading and writing, in addition to difficulties in understanding some of the Kiswahili terms. Moreover, the workshop sessions were not recorded for the comfort of the participants. Thus, it was not possible to complete a robust and external fidelity check.

### 4.6. Future directions

In future trials of this workshop, it could be insightful to get a larger sample size and more long-term follow-up to determine if and how the findings of this study vary. Collecting data from the children of participants would also be beneficial to find out whether improvements in participants’ perception of family relationships are reflected in children’s responses. Additionally, implementing this workshop in tandem with the youth focused Shamiri Intervention would help determine if and how the impact of each program is affected by the other.

## Data availability statement

The raw data supporting the conclusions of this article will be made available by the authors, without undue reservation.

## Ethics statement

The studies involving humans were approved by Kenyatta University Ethics Review Committee. The studies were conducted in accordance with the local legislation and institutional requirements. The participants provided their written informed consent to participate in this study.

## Author contributions

RA came up with the initial concept and study design and designed and oversaw the needs assessment phases. RA, HN, RB, VN, TO, and CW refined the study design and implementation method. RA and RB curated the study measures, while RA developed the original measures. HN coordinated and participated in the translation of all study measures. RA and VN designed and implemented the training for workshop facilitators. RA, HN, RB, and VN coordinated implementation. RB and TO conducted quantitative analysis, while RA conducted qualitative analysis. All authors contributed to this manuscript and approved of the final version.

## Funding

This study was funded by Shamiri Institute, a Kenya – based mental health nonprofit committed to developing and disseminating youth mental health interventions.

## Conflict of interest

The authors declare that the research was conducted in the absence of any commercial or financial relationships that could be construed as a potential conflict of interest.

## Publisher’s note

All claims expressed in this article are solely those of the authors and do not necessarily represent those of their affiliated organizations, or those of the publisher, the editors and the reviewers. Any product that may be evaluated in this article, or claim that may be made by its manufacturer, is not guaranteed or endorsed by the publisher.
