# Supplementary material for: Group-based psychoeducational workshop for parents in Kenya: findings from a pilot study
Source: Front Public Health. 2023 Sep 15;11:1223804. doi: 10.3389/fpubh.2023.1223804 (PMC10541024; doi:10.3389/fpubh.2023.1223804)
Supplement: Supplementary file 1 [file Data_Sheet_1.docx]

**SHAMIRI PARENT PSYCHOEDUCATIONAL WORKSHOP**

**WORKSHOP PROTOCOL FOR GROUP FACILITATORS**

**Workshop Schedule**

- **Required Materials:**
  - Baseline questionnaires
  - Endpoint questionnaires
- **Workshop Overview:**
  - Part 1 : Introductions and Baseline Questionnaire – *20 minutes*
  - Part 2 : Module 1 – Importance of Parental Support – *15 minutes*
  - Part 3 : Module 2 – Kenyan Youth Mental Health – *15 minutes*
  - Intermission – *10 minutes*
  - Part 4 : Module 3 – Shamiri Intervention – *40 minutes*
  - Part 5 : Module 4 – Psychological First Aid – *10 minutes*
  - Part 6 : Conclusion and Endpoint Questionnaire – *10 minutes*

**Part 1: Introductions & Baseline Questionnaire (20 minutes)**

*Note for facilitator: Introduce yourself and ask all participants to introduce themselves.*

As some of you may know, Shamiri’s main purpose is to design and provide sustainable and culturally appropriate solutions to improve student wellbeing. Until now, our focus has been working directly with secondary school students. However, we have realized the importance of working with parents, teachers, and other important stakeholders in the lives of students.

As a result, moving forward, we plan to collaborate with parents and teachers to improve the impact of our project on students' mental wellbeing, and on the community in general.

The **purpose of this workshop** is to cooperate and support each other with the shared goal of improving student wellbeing in Kenya.

In this workshop, I will share what we have learnt from our experiences working with students. I will ask you a few questions because we want to learn from your experiences as parents. We will also talk about the 4-week Shamiri project for students and how the main lessons of the project can be helpful for students and for families.

**Baseline data collection.** We want to make sure that this workshop achieves these goals. So, we have put together a set of questions for you that will help us collect data and to determine if and how this workshop helps.

*Note for facilitator: hand out baseline questionnaires to participants.*

So, before we start the discussion, please answer these questions. Do not worry about answering them correctly; there is no right or wrong answer. Your answers will remain confidential, and your identity will not be shared with anyone outside of the Shamiri team. At the end of the session today, we will have another set of questions to get your feedback on this workshop.

**Part 2: Module 1 – Importance of Parental Support (15 minutes)**

All of us might be aware that parental support is incredibly important and necessary for student wellbeing. This is especially for students between the ages of 13 to 18 since they are going through many changes and transitions in life.

Question: In your opinion, what are some of the most important ways a parent can support students in this developmental period?

*Note for facilitator: after participants discuss their answer, please share the following as additional/supportive information to what they have shared (if they have not mentioned it already).*

In previous projects that Shamiri has done with Kenyan students, we have collected a lot of data/information because we wanted to be sure that the project was helping to improve student wellbeing. We also used this data/information to better understand student wellbeing, and specifically their mental health.

- Mental health is a state of well-being whereby one realizes his or her own abilities; copes with the normal stresses of life, works productively and can contribute to their community.

We found that the students who felt that they received good social support from their family and friends were likely to have better mental health. We also found that students who got less social support from family and friends were likely to have poorer mental health.

Question: While we all know parent support is important for students, we also know that parents might need additional support for their own wellbeing and to allow them to support their students. As parents, what kind of support would you like?

**Part 3 : Module 2 – Kenyan Youth Mental Health (15 minutes)**

Question: Based on your experiences and the definition of mental health, what are the problems that students are facing?

*Note for facilitator: after participants discuss their answer, please share the following as additional/supportive information to what they have shared (if they have not mentioned it already.)*

Students explained that they feel pressured by parents and the school to perform well academically and are given a significant amount of schoolwork. Schools have high academic expectations and focus heavily on academics, often at the cost of social and emotional wellbeing.

In the past year, for example, students faced a significant amount of schoolwork with limited time to complete it. Students also mentioned that parents tend to compare them either with other students or with the parents’ own upbringing, which sometimes causes rifts in family relationships.

Question: Based on your experiences, how do you think these problems such as academic pressure, feeling that they are being compared, having no one to talk to affect students’ mental wellbeing?

*Note for facilitator: please share the following with the parents as additional/supportive information to what they have shared if they have not mentioned it already.*

When the Shamiri team conducted Shamiri program with the students in 2019, we also collected particularly important data from the students. Some of this data focused on mental health challenges faced by the students.

Our questions measured mental health challenges such as problems with sleep, feeling sad, feeling hopeless, problems with concentration, and so on. Other questions we asked measure factors that affect mental health such as excessive worrying, feeling restless, being easily irritable, and so on.

These behaviors, at low levels, might sound familiar to everyone. However, when someone experiences them in elevated levels and for a long/extended period, they become mental health challenges. Based on our results from these questions, about half of the students had scores which were high enough to indicate that they needed mental health support.

*Note for facilitator: leave room for parents to reflect on this information.*

**INTERMISSION – 10 MINUTES**

**Part 4 : Module 3 – Shamiri Intervention (40 minutes)**

Question (3 minutes): What would you suggest as potential solutions for better youth mental health?

*Note for facilitator: transition into the lesson by framing it like ‘thank you for sharing your solutions. Now, I will share with you our solutions that we have tested and found that they can be helpful to students with mental health challenges.’*

**Introduction to the Shamiri Intervention (10 minutes)**

The purpose of the Shamiri Intervention is to improve Kenyan students’ mental health. High school students who have participated in this intervention experienced an improvement in their academic grades and social relationships and a decrease in mental health challenges.

For this intervention, participating students meet once a week for four weeks in groups of 8-10 other students. These groups meet in schools and are led by local high school graduates who have been trained by the Shamiri team.

Since this intervention focuses on character building and mental wellbeing, it makes it easy for the students to participate without facing any stigma. The components of the Shamiri intervention teach students about having a growth mindset, expressing gratitude, and working on their personal values.

**Components of the Shamiri intervention.** In the growth-mindset sessions, students learn that personal characteristics can be changed and are not fixed at birth. They learn that even the brain itself can grow and change based on the person’s activities and habits. The students also learn about problem solving techniques and how to use these when they face challenges in various aspects of life. e.g., academic challenges and social relationships.

In the Gratitude session, students learn to recognize and appreciate the positive experiences in their lives. This lesson is important because practicing gratitude in this manner helps improve one’s wellbeing and life satisfaction. This concept also helps students develop a habit of applying gratitude in their personal life, their social interactions and school life.

In the Value affirmations session, students reflect on their most important values. They then learn how to use these values to set their own personal goals and develop their problem-solving skills. These lessons and activities can help them feel more purposeful.

Question: Any initial comments or questions?

Question: In your opinion, what are the best parts of this Shamiri program?

Question: Do you think this Shamiri program can help students? How so?

Question: What parts can be improved (added or removed) to make this program even more helpful for the student?

**Components of Shamiri Program and Family Relationships (20 minutes)**

We will now explore how to incorporate some of the concepts we discussed into conversations between parents and adolescent children and how doing this can benefit students and parents as well.

**Growth mindset.** For students, having a growth mindset and believing that it is possible to change their old habits and develop new skills can give them hope. It can also make them want to try and improve their life in different ways.

- For example: Let’s say a student is doing poorly in their mathematics class. They do not like the class; they do not understand it; and they are failing their tests. If this student learns that they can develop new skills that can help them do better in mathematics class, this can give them hope to try to grow and improve.
- In addition to learning about growth mindset in the Shamiri Intervention, it is also important for the people closest to the student to also learn about this concept. If their parents and their teachers also believe that people can grow and work on new skills, it would be really encouraging for the student.

**Fixed mindset*.*** The opposite of a growth mindset is a fixed mindset. A person who has a fixed mindset might believe that people are born with a set of characteristics that don’t change. If people believe that they cannot change, then they probably will not try to change and improve their behavior or work on new habits.

- For example: Let’s say a student does not participate in class very much. There might be many reasons for why this student is not participating in class. If the student and their parents and teachers believe that not participating is in this student’s nature, they might label this student as quiet or lazy. And if the student believes this and feels like there is no hope for improving their behavior, then they would not try to change.
- Therefore, it is important to know that change is possible and, for students, to have parents and teachers who know that they can grow and encourage them to change for the better.

Question: Can you identify some social or cultural practices that encourage having a fixed mindset?

Question: Similarly, what are some of the things you can do to help your children avoid having a ‘fixed mindset’ and encourage the ‘growth mindset’?

**Growth mindset and gratitude.** Since practicing gratitude has benefits for one’s wellbeing, it is important to help students feel and express gratitude. Helping students be grateful for themselves and their own efforts at growing and improving can also be beneficial.

- For example: Let’s say a student failed their math class last term. This term, the students had been trying to listen in class more and study more, and they received a D. This might seem like a small amount of growth, but it is still positive. If the student’s parents encouraged this growth, it could help the student feel grateful for their parents’ support and for their own efforts. It could also motivate them to keep trying to improve their grades and understand that growth takes time.

**Value affirmations.** When raising and teaching children, it can be common to punish unwanted or negative behaviors. This might teach students that the important thing is to avoid punishment by avoiding or hiding these unwanted behaviors.

- For example: Let’s say a student often talks with their classmates during class and disturbs the teacher. At the end of each term, the teacher writes a note about the student’s bad behavior in class and asks the student to get their parents to read and sign the note. The first two times the student showed the note to their parents, they got punished for their bad behavior and told to never bring a note like that home again. So, the next time the student gets a note like that, they will just fake their parents’ signature and take it back to the teacher. And they might feel that they have successfully faced this problem since their main goal is to avoid being punished and not necessarily to be well behaved in the classroom.
- But, in a different scenario, this student’s parents explained to them the harmfulness of disturbing in class and encouraged the student to be well-behaved in the classroom. The parents also told the student that if they tried to be more well-behaved in the class and if they bring a teacher’s note about their improved behavior at the end of the term, they will get a reward. The reward can be that the parents will cook the student’s favorite meal or that the student gets to avoid doing chores for a week or something else the student really wants. This can make the student want to improve and want to grow, instead of just figuring out a way to avoid punishment.

**Activity (7 minutes).** In this activity, I will read a small paragraph describing a situation a student is in. As I am reading, try to imagine yourself as the parent of this student.

*Chumba is a Form 4 student at a day school. While she was in Forms 1-3, she always tried to listen in class and study when she got home. However, she often got really stressed and nervous before tests and exams. Even while sitting for exams, she would get so stressed that she would not be able to focus on the exam questions. As a result, her grades are usually below average, and her teachers and family always pick on her for being lazy and not bright. Now, as a Form 4 student, she is feeling really stressed about the KCSE (Kenya Certificate of Secondary Education). Just the act of being in school has been making her feel nervous so she has been cutting classes. She would put on her uniform and leave the house as if she were going to school but end up meeting up with her friends who are also cutting school. Being with her friends and avoiding getting called lazy or stupid helps her feel relaxed. After a week of Chumba not showing up to school, you (her parents) get a phone call from Chumba’s school about her poor attendance.*

With the person next to you, discuss how you/a typical Kenyan parent might have reacted to this situation before this workshop and how you would react based on what you have learnt in this workshop.

*Note for facilitator: ask the following question after allowing parents to discuss with each other.*

Question: How would you have handled the situation after the call from the school? Please share some of what you discussed with the group.

***Part 6: Module 4 – Psychological First Aid (10 minutes)***

Psychological First Aid is the support or assistance one person can offer to another who may need some support after experiencing a distressing event. More specifically, this type of support can be provided by parents to their children if they are going through a tough time.

People who can benefit from receiving PFA include a person who has disclosed about a crisis they are going through. The crisis can range from having failed an exam to having been sexually abused to experiencing suicidal thoughts and feelings.

Note that not everyone who goes through a crisis will need or want PFA as people respond to crisis differently. Make yourself available to those who want your support but do not pressure those who do not want your support.

PFA can be provided by anyone; you do not have to be a professional. It entails:

- choosing a safe place that you are both comfortable with,
- listening empathetically and calmly
- comforting the person in need to make them feel safe,
- protecting them from further harm,
- assisting them to access additional/supportive/supportive social, physical, or emotional support if needed.

**Confidentiality.** In situations where you are providing PFA to students who are not your own students, it is important for the affected person to know that you will not share their sensitive story with others. Keeping their story private allows the person to feel free to express themselves and builds their trust in you. This protects the affected person from the stigma they might receive from others which can cause further psychological distress.

However, there are times you can contact others for help. These include when the affected person is at risk of hurting themselves or others. Examples are:

- If they express plans to attempt suicide
- If there is evidence of significant abuse or neglect
- If there is evidence of life-threatening drug use

If any of these examples occur, it could be beneficial to contact a mental health professional, like a counselor or psychologist, since they will be able to provide additional/supportive/supportive and professional support for the person.

Always discuss with the affected person before contacting someone else and clearly explain the reasons why you must do this. This may look something like: “I have a responsibility to make sure that you’re not at risk of harm so I may have to share this information with someone who will help keep you safe.”

Question: Any initial thoughts, comments, or reactions?

Question: What is the most interesting or surprising thing you’ve heard from our discussion so far?

Question: In the African context, how do we usually offer support to others who are in crises?

Question: Do you agree that PFA would be important for students? Why do you think so?

**Part 6: Conclusion and Endpoint Questionnaires (10 minutes)**

Question: Any final thoughts, comments, or reactions?

**Endpoint questionnaires.** These are questionnaires that are like the ones you filled out in the beginning. Just as a reminder, we will be using the data to determine if and how this discussion helped. Your personal information will remain confidential.

*Note for facilitator: hand out endpoint questionnaires.*

**Follow-up data collection.** We will also ask you to answer these questions again after one month and compare your answers with today’s. Also, if you are willing, after two-weeks, we will call you for a short conversation about the workshop and whether it has impacted your family relationships.
