# Supplementary material for: Group-based psychoeducational workshop for parents in Kenya: findings from a pilot study
Source: Front Public Health. 2023 Sep 15;11:1223804. doi: 10.3389/fpubh.2023.1223804 (PMC10541024; doi:10.3389/fpubh.2023.1223804)
Supplement: Supplementary file 2 [file Data_Sheet_2.docx]

**Shamiri Institute – Parent Psychoeducational Workshop**

**Needs Assessment Report – Phase 1**

Table of Contents

[Objectives 1](#_Toc125625374)

[Methodology 1](#_Toc125625375)

[Student Focus Group Discussions 1](#_Toc125625376)

[Results 2](#_Toc125625377)

[Conclusion 3](#_Toc125625378)

[Parent Focus Group Discussion 3](#_Toc125625379)

[Results 4](#_Toc125625380)

[Conclusion 5](#_Toc125625381)

[Implications 5](#_Toc125625382)

# Objectives

The primary objective of this needs assessment phase was to understand the barriers to communication between students, parents, and teachers. This was a priority since our team had observed strained relationships between these parties during previous research projects. The secondary objective of this needs assessment was to identify potential solutions to improve these relationships and overall youth wellbeing.

# Methodology

Our team conducted a series of focus group discussions with secondary school students, parents, secondary school teachers, and school administrators. More details regarding participant demographics of the student and parent FGDs are provided in the subsequent sections.

All FGDs lasted approximately one hour each and were conducted by members of the Shamiri Institute team. The sessions began with a few ice breakers before explaining the purpose of the discussion and the roles of the moderator and assistant moderator. The moderator instructed the participants to read and sign the informed assent or consent forms and obtained verbal consent to record the discussion. Participants then discussed the questions posed by the moderator while the assistant moderator noted responses and monitored the audio recording. For the last few minutes of the session, the assistant moderator presented a summary of the discussion to the participants and the moderator instructed them to complete brief feedback forms. Participants were then given T-shirts/coffee mugs and pens.

The notes and audio recordings were referred to when conducting a thematic analysis to identify codes amongst the students’ responses. Each code was a short phrase which represented an independent idea, concern and/or suggestion which came up in the FGDs. These codes were primarily categorized into themes. In this case, most themes are equivalent to the questions asked by the moderator. Within each theme, similar codes were categorized into sub-themes for more efficient analysis and reporting processes. The audio recordings and notes were then used to calculate the frequency of each code and theme. Both the code frequency and the notes from the assistant moderators were considered when finalizing the results.

# Student Focus Group Discussions

The purpose of this set of FGDs was to get students’ perspectives of the following: (a) factors affecting youth wellbeing, (b) factors affecting the youth’s relationship with parents and schools and (c) new and existing solutions to improve youth mental wellbeing.

Three focus group discussions (FGDs) were conducted at three schools in and around Nairobi: Highway Secondary School and Ofafa Jericho High School, both all-boys’ boarding schools, and Kanjeru Girls Secondary School, an all-girls’ day school. Participants in the FGDs were selected randomly by teachers. There were between 6-7 students in each group which represented Forms 1-4 and were randomly selected by the guidance and counseling teacher or the deputy principal.

## Results

For each theme, the sub-themes and codes with the highest popularity amongst the FGD participants are listed in descending order.

**Theme 1: Factors Affecting Student Wellbeing**

Participants highlighted academic pressure as the primary factor affecting student wellbeing. They explained that students feel pressured by both parents and the school to perform well academically, in addition to being given a significant amount of schoolwork. Participants further explained that schools have high academic expectations and focus heavily on academics, often at the cost of social and emotional wellbeing.

**Theme 2: Factors Affecting Student-Parent Relationships**

Participants agreed that lack of trust was the main challenge to student-parent relationships. They explained that parents are very controlling and often treat them like young children. Academic pressure from parents also contributes to a rift in their relationships. Participants stated that parents tend to compare their children with other children, without acknowledging their children’s unique set of capabilities. They also explained that parents tend to compare their upbringing with that of their children, which according to the participants, is often irrelevant and unpleasant. Additionally, participants in the day school stated that they were overwhelmed by the number of chores they were given while participants in the boarding schools explained that their relationships with their parents were strained by the lack of time spent together.

**Theme 3: Factors Affecting Student-Teacher Relationships**

Participants raised several issues with school administrators and teachers, many of which regarded teachers. They explained that teachers often compare students, ridiculing those who don’t perform well academically, and favoring those that do. Participants also stated that teachers are often unfriendly and unapproachable by students.

**Theme 4: Stressors in School Environment**

Participants agreed that peer pressure is a significant stressor in school environments. They explained that students often influence their peers regarding their subject selection and drug use. Participants also stated students from low-income families face additional stressors when comparing themselves with students who have allowances. Additionally, participants attending boarding schools highlighted that schools have limited resources (food, water) and do not allocate enough time for students to sleep.

**Theme 5: Thoughts on Prevalence of Mental Health Problems Amongst Kenyan Youth**

In this section, the moderator shared data regarding Kenyan youth mental health, highlighting that 1 in 2 Kenyan youth are likely to experience various symptoms of depression and anxiety. Most participants expressed agreement with the results and explained that academic pressure causes stress amongst students. Moreover, participants stated that problems at home and lack of support from parents causes sadness and problems with concentration amongst students.

**Theme 6: Common Coping Strategies Amongst Youth**

Participants emphasized that drug use was the most common coping strategy amongst youth. They explained that most students start using drugs due to peer pressure and that they continue to do drugs to feel happier and boost concentration. Additionally, participants highlighted that male students often keep to themselves while female students tend to ask their friends for advice and immerse themselves in hobbies. Participants also explained that even though some students go to the school counselors for help, most students are concerned that school counselors -who also work as teachers- might share their personal information with other teachers.

**Theme 7: Potential Solutions to Improve Youth Wellbeing**

Participants cited a need for programs that support students to develop skills that can be used outside of the classroom. Participants also stated that they would like for teachers to be more friendly and motivating towards students. Moreover, participants explained that they would also like reduced schoolwork and increased time to relax while in schools. Finally, participants stated that schools could benefit from increased financial resources to fund improved student facilities and to host recreational events.

## Conclusion

Across the student FGDs, the participants explained that the primary factor affecting their wellbeing is academic pressure, both from parents and teachers. Participants also explained that they feel as though parents do not trust them and neglect to acknowledge their own unique set of capabilities. Moreover, students expressed their desire for increased time spent with parents.

# Parent Focus Group Discussion

The purpose of this FGD was to get parents’ perspectives on the following: (a) factors affecting youth wellbeing, (b) factors affecting parents’ relationships with their children and their schools and (c) new and existing solutions to improve youth mental wellbeing.

This focus group discussion (FGD) was conducted with Kenyan parents who were invited to the Shamiri Campus. Amongst the 7 participants, 4 were female. The participants had children who were attending the following Kenyan Secondary schools: Maryhill Girls High school, Limuru Girls High School, Moi Educational Centre High School, Premier Academy, Mount Carmel Secondary School & Logos Christian school.

## Results

For each theme, the sub-themes and codes with the highest popularity amongst the FGD participants are listed in descending order.

**Theme 1: Factors Affecting Youth Wellbeing (School-related)**

Amongst the school-related factors, schools being too crowded and having a heavy workload with limited time for relaxation were emphasized by participants. They explained that schools which don’t allow individuality amongst students and teachers who are stressed and/or overworked were also important factors affecting student’s wellbeing. Other factors that were mentioned include the high academic pressure tied to the KCSE and peer pressure.

**Theme 2: Factors Affecting Youth Wellbeing (Family-related)**

Among the family-related factors, the issue of lack of guidance for students was the most important for participants. They explained that youth wellbeing is significantly impacted when they don’t receive the necessary guidance at home, especially during critical times like the transition to secondary school. Other family-related factors that were mentioned included parental wellbeing, as well as the socioeconomic and marital status of parents. Participants also explained that the COVID-19 pandemic has led to increased academic pressure on students and worsened parent-teacher relationships.

**Theme 3: Factors Affecting Parent-Teacher Relationships**

Participants highlighted lack of respect as the most significant issue affecting parent-teacher relationships. They explained that, on one hand, there are parents who look down on the teaching profession and treat teachers in a disrespectful manner. On the other hand, there are teachers who look down on students and their parents due to their low socioeconomic status. Participants also pointed out that “the blame game” was another factor affecting parent-teacher relationships; whenever a student is facing an issue, parents tend to blame the teachers while teachers blame the parents.

**Theme 4: Factors Affecting Parent-Child Relationships**

Much of this discussion focused on parents’ wrongdoings, with an emphasis on the following: not allowing individuality and vulnerability at home, “tyrannical parenting”, not having good parenting skills, and having unrealistic expectations for children. Participants explained that having a “nothing new under the sun” mindset has also impeded parent-child relationships since it encourages parents to assume the desires and needs of their children as opposed to being inquisitive and keeping an open mind. Another factor affecting parent-child communication was a generational barrier. Participants explained that parents often feel very out of touch regarding the music, technology and language used by their children and find it difficult to adapt their parenting skills to fit their children’s specific needs. Additionally, participants also stated that children who don’t listen to parents and the gradual decrease in community parenting has negatively impacted parent-child relationships.

**Theme 5: Thoughts on Prevalence of Mental Health Problems Amongst Kenyan Youth**

In this section, the moderator shared data regarding Kenyan youth mental health, highlighting that 1 in 2 Kenyan youth are likely to experience various symptoms of depression and anxiety. Almost all participants expressed that the data matched their expectations.

**Theme 6: Solutions to Improve Youth Wellbeing**

Participants shared ideas which mostly highlighted ways in which parents could provide better support to their children. They explained that children would benefit from parents allowing more individuality and listening more instead of assuming their children’s needs. Participants also mentioned that parents would benefit from increased access to resources for improving parenting skills. Another potential solution offered was breaking restrictive traditional gender norms to mitigate the fact that girl children are raised to be more afraid of failure and risk-aversive. Participants also added that addressing the high academic pressure in schools would also be quite beneficial to improve youth wellbeing.

## Conclusion

Throughout the discussion, participants were quite introspective and identified numerous parental wrongdoings which were negatively impacting youth wellbeing. It was clear that participants were quite concerned about youth wellbeing and willing to play a more active role to promote their children’s wellbeing.

# Implications

The objectives of this needs assessment phase were (1) to have a better understanding of challenges in communication between students, parents, and teachers, and (2) to identify potential pathways to address these challenges and improve youth mental wellbeing. The findings indicate that there is a need for parents (and teachers) to become aware of challenges faced by students and how they might be initiating/exacerbating these challenges. There appears to be lack of understanding between these stakeholders which is significantly contributing to barriers in communication. The findings also indicate that these challenges can be alleviated by providing parents with resources that can foster their skills in supporting and communicating with students. Such resources could improve not only the relationships between these stakeholders, but also student mental wellbeing.
