# Supplementary material for: Group-based psychoeducational workshop for parents in Kenya: findings from a pilot study
Source: Front Public Health. 2023 Sep 15;11:1223804. doi: 10.3389/fpubh.2023.1223804 (PMC10541024; doi:10.3389/fpubh.2023.1223804)
Supplement: Supplementary file 3 [file Data_Sheet_3.docx]

**Shamiri Institute – Parent Psychoeducational Workshop**

**Needs Assessment Report – Phase 2**

Table of Contents

[Objective 1](#_Toc132367421)

[Feedback from General Population 1](#_Toc132367422)

[Methodology 1](#_Toc132367423)

[Results 2](#_Toc132367424)

[Conclusion 2](#_Toc132367425)

[Feedback from Target Population 2](#_Toc132367426)

[Methodology 3](#_Toc132367427)

[Results 3](#_Toc132367428)

[Conclusion 4](#_Toc132367429)

[Implications 4](#_Toc132367430)

# Objectives

Based on findings from the first phases of needs assessment and workshop design, a range of options were developed for implementation method and workshop content. The options for implementation method consisted of the following: a virtual workshop, two different hybrid workshops, and an in-person workshop. The options for workshop content consisted of modules focused on the following topics: Psychopathology, Psychological First Aid, and Shamiri Intervention. The introductory module was not included in this phase of needs assessment as the study team agreed on its crucial role to set up the discussion for the remaining modules.

The purpose of this phase of needs assessment was to identify the options which are most popular amongst Kenyan parents of adolescents. These options were to determine the outcome of the second phase of the workshop design.

# Feedback from General Population

In this phase, our team sought to collect data from parents which represent the general Kenyan population. Accordingly, we arranged to work with Alliance High School (AHS), a National high school with students from different parts of the country.

## Methodology

Initially, our team designed brief questionnaires which would allow us to identify parents’ most and least preferred options for workshop content and implementation method. These questionnaires were distributed to parents attending an AHS Annual General Meeting. During this meeting, the purpose of the overall study and the questionnaire were explained and all attending parents were invited to complete the questionnaire. Approximately 150 parents —roughly a third of all parents in attendance— completed the questionnaire.

All analyses were done using R Studio (version 4.3.0). For each question —asking for parents’ most or least preferred option— a chi-square test was used to determine if the preferences for each option were significantly different from each other.

## Results

Results of the chi-square tests for the relevant questions are available below.

**Question 1: Most Preferred Module**

Approximately 50% of the participants opted for the Shamiri Intervention module. The Psychoeducation module and the PFA module were the second and third preferred, respectively. The differences in preference were significant (p<0.001) amongst all options.

**Question 2: Least Preferred Module**

72% of the participants indicated that the PFA module was their least preferred. The Psychoeducation module and the Shamiri Intervention module were the second and third least preferred, respectively, complementing the results from the previous question. The differences in preference were significant (p<0.001) amongst all options.

**Question 3: Most Preferred Implementation Method**

The first of the hybrid options, the virtual workshop complemented by an in-person discussion was selected by 40% of the participants. The second of the hybrid options, the virtual workshop and the in-person workshop were reported to be the second, third and fourth most preferred options, respectively. The differences in preference were significant (p<0.001) amongst all options.

**Question 4: Least Preferred Implementation Method**

Over 50% of the participants selected the in-person workshop as their least preferred implementation method. The virtual workshop, the second of the hybrid options, and the first of the hybrid options were reported to be the second, third and fourth least preferred options, complementing the results to the previous question. The differences in preference were significant (p<0.001) amongst all options.

## Conclusion

The purpose of this questionnaire was to determine parents’ most preferred options for workshop content and implementation methods. Our analyses strongly indicated that these options are the Shamiri Intervention module and the first of the hybrid options—a virtual workshop followed by an in-person discussion. The least preferred options were the Psychological First Aid module (PFA) and the in-person workshop.

# Feedback from Target Population

To supplement the feedback from the general population, our team sought to collect data from parents in a similar demographic as the target population. If the findings from the target population differed from those of the general population, the former were to be prioritized in the subsequent phase of workshop design.

## Methodology

Our team set up a focus group discussion (FGD) with six parents whose children attend Elite Visionary High School —a school located in Kibera, Nairobi. The FGD participants were randomly recruited by a teacher at the school.

The FGD lasted ~60 minutes and was conducted in Kiswahili by two staff members. After personal introductions were made, the discussion began with an explanation of the purpose of Shamiri Institute and the discussion. The moderator then explained the purpose of the informed consent forms and went over some group norms. After participants signed the consent forms, the moderator presented the different options for implementation method and workshop content. While the participants discussed these options, the assistant moderator took notes on their feedback. After the discussion was concluded, the assistant moderator shared their highlighted notes with the participants.

After the FGD, the moderator and assistant moderator met with the PI to discuss their takeaways from the discussion. These takeaways and the assistant moderator’s notes were used for the extraction of codes -a short phrase/sentence representing a stand-alone idea/thought. These codes were primarily categorized into themes. In this case, most themes are equivalent to the questions asked by the moderator. Within each theme, similar codes were further categorized into sub-themes for more efficient analysis and reporting processes. The audio recordings and notes were then used to calculate the frequency of each code and theme. Both the code frequency and the notes from the assistant moderators were taken into account when finalizing the results.

## Results

For each theme, the sub-themes and codes with the highest popularity amongst the FGD participants are listed in descending order.

**Theme 1: Feedback on Psychoeducation Module**

Participants expressed that this module would be helpful for parents and their families by providing support during economic hardship. They explained that, for families like theirs, financial constraints have resulted in strained parent-child relationships, and that these relationships would be improved by parents being able to communicate with understand their children better. However, after all the options for workshop content were discussed, only one participant chose this module as their preferred module.

**Theme 2: Feedback on the PFA Module**

Participants stated that this module would be helpful for parents by helping them avoid worsening their children’s emergencies/crises. They agreed that it is common for parents to constantly reprimand their children during such emergencies and that this module would provide alternative ways to respond. Participants also agreed that this module would be beneficial for families living in poverty. However, like the previous module, only one participant chose this module as their preferred one.

**Theme 3: Feedback on the Shamiri Intervention Module**

Participants agreed that learning about the concepts of the Shamiri Intervention -growth mindset, gratitude, and values- would allow parents to improve their relationships with their children. They explained that these concepts would encourage parents to be more encouraging and appreciative and to believe in their children’s ability to grow. Four of the six participants chose this module as their preferred one.

**Theme 4: Feedback on In-Person Implementation**

Participants stated that in-person discussions -with trained professionals as facilitators- would be beneficial. They also agreed that the workshop should not have multiple sessions and should be held in a convenient setting like their children’s school. After discussing all the implementation methods, all participants chose this method as their preferred one.

**Theme 5: Feedback on Digital and Hybrid Implementation**

Even though participants expressed interest in digital tools such as audio recordings, they all agreed that such tools would be inaccessible to most parents like themselves. They explained that this is due to limited access to smart phones and inadequate funds for internet access. As an alternative, participants suggested using text messages (SMS) to share summaries of in-person workshops with parents who were absent from these workshops.

**Theme 6: General and Miscellaneous Feedback**

All participants expressed general approval of the workshop and strong interest in participating in such a workshop. Participants also stressed that some parents might be reluctant to receive lessons which might appear as criticism from strangers. As additional features, participants suggested the payment of school fees (for participating parents) and including members of clergy during implementation.

## Conclusion

The purpose of this FGD was to identify the preferences of parents in our target population. Based on the findings from discussion, we have determined that parents in our target population are likely to prefer the Shamiri Intervention Module and the in-person implementation method. This FGD highlighted the need to consider parents’ reluctance to attend workshops if they anticipate receiving criticism during the final design of the workshop content and training for facilitators. Even though our workshop is not capable of accommodating these elements, this FGD also highlighted that parents would be interested in financial support and inclusion of religious components in the workshop.

# Implications

The purpose of this phase of needs assessment was to narrow down the options for implementation method and workshop content based on parents’ preferences. Accordingly, our team collected data from samples of the general and target parent population. As mentioned previously, if the findings from the two groups differed, the findings from the target population were to be prioritized in the final stage of workshop design.

In terms of workshop content, both groups identified the Shamiri Intervention module as their most preferred option. Therefore, this module was to be allocated the most content and discussion time as compared to the remaining two modules. In terms of implementation method, the most preferred option differed between the two groups. Here, the study team decided to proceed with the in-person implementation method considering that it was the preference of the target population sample and the limited access to smart phones and inadequate funds for internet access amongst this population.
